# Nudging socially isolated people towards well-being with the ‘Happiness Route’: design of a randomized controlled trial for the evaluation of a happiness-based intervention

**DOI:** 10.1186/1477-7525-11-159

**Published:** 2013-09-20

**Authors:** Laura A Weiss, Gerben J Westerhof, Ernst T Bohlmeijer

**Affiliations:** 1 Faculty of Behavioral Sciences, Psychology, Health & Technology, University of Twente, P.O. Box 217 7500 AE, Enschede, Netherlands

**Keywords:** Positive psychology, RCT, Happiness-based approach, Nudging, Low SES, Health problems, Well-being, Loneliness, Purpose in life, Resilience

## Abstract

**Background:**

The Happiness Route is an innovative intervention that uses a happiness-based approach for people with an accumulation of risk factors for low well-being: socially isolated people with health impairments and a low socioeconomic status. The goal of this intervention is to improve well-being by engaging participants in intrinsically motivated activities with methods from positive psychology. We hypothesize that the primary outcome measure, emotional, social and psychological well-being of participants of the Happiness Route, will increase in comparison to the traditional and commonly-used problem-based approach. Secondary outcome measures are health-related quality of life, psychosocial functioning and health care consumption.

**Methods and design:**

Participants will be socially isolated people with health problems and a low socioeconomic status. Participants will be recruited in ten Dutch communities and candidates will be signed up by intermediaries, professionals from the health and social sector. Randomly assigned, half of the participants will follow the Happiness Route and half of the participants will follow the active, problem-focused control group ‘Customized Care’. In total, 256 participants will be included. In both conditions, participants will receive counseling sessions from trained counselors. In the control group, participants will talk about their problems and the care they get and counselors help to optimize their care. In the Happiness Route, the counselor ask questions such as “How do you want to live your life?”. The intervention helps people to find their ‘passion’, i.e., a positive goal-engaged and intrinsically motivated activity. It enables them to follow their passion through by a once-only personal happiness budget (maximal €500). We use well-validated and reliable questionnaires to measure primary and secondary outcome measures at baseline, directly after the intervention and at a nine-month follow-up.

**Discussion:**

Shortcomings of earlier intervention studies in positive psychology will be tackled with this study, such as having a target group who is especially vulnerable for low well-being. The practice-based setting is especially interesting, as it can give valuable insights in how positive psychology interventions work in practice, but can also give rise to several challenges.

**Trial registration:**

Dutch Trial Register, trial registration number TC=3377NTR.

http://www.trialregister.nl/trialreg/admin/rctview.asp?TC=3377.

## Background

In the health care system, the problem-based approach is widely used: the goal is to diagnose and treat the health problem. Healthcare and social workers are trained in this problem-based working style. This approach works well for most people. Yet there is a group where this approach does not seem to work. This group has an accumulation of risk factors for low well-being; severe feelings of loneliness, health problems and a low socioeconomic status (SES) [1-5]. These factors may reinforce each other.

One of the factors that contribute most to well-being is a rich social live [6]. But this is not given for everyone. People with health problems and a low SES are at high risk for social isolation. Hence this group suffers from different social, health and financial problems and low well-being. The traditional problem-based approach can only provide them with more and more care [7]. Accordingly, this will lead to increased economic costs for the society as well as for the individual [8].

An alternative approach is needed for this target group, one that focuses on promoting positive mental health by stimulating happiness, self-realization and social integration [9]: a happiness-based approach. In the Netherlands, this is recently acknowledged in public health, where changes in welfare policies towards individual responsibilities and self-management of citizens have been encouraged [10-12]. The Dutch Council for Public Health and Health Care [12] has recently advised the Ministry of Health to shift the focus in health care from ‘illness and care’ towards ‘behavior and health’. These changes ask for a more positive focus instead of the traditional problem-focused approach and interventions. The patients’ problems should not be the only subject of interest, but their well-being also has to be acknowledged.

Mental well-being, broadly defined as emotional, social and psychological well-being, has many positive effects. Besides the fact that most people strive for happiness in their lives, it also improves many aspects of health and longevity [13-15] and personal functioning, such as productivity [14]. Interventions directed to improve well-being seem to have positive effects [16]. Meta-analyses show that the promotion of well-being will lead to considerable health gains for the individual and society [13,17-20].

Still, there are only few projects in the Netherlands that make use of this new happiness-based approach. The ‘Happiness Route’, a short behavioral intervention to promote positive mental health, is one of the few that is directly targeted to improve well-being. This innovative intervention uses insights from positive psychology and a recent theory from behavioral economics, namely ‘nudging’: giving people a gentle push in the ‘right’ direction [21,22]. Accordingly, the aim of this short behavioral intervention is to increase well-being by nudging people towards an intrinsically motivated activity. To be able to conduct this activity, participants receive a once-only budget with a maximum of €500.

The Happiness Route is based on a theory that is often used in positive psychology: the self-determination theory. Deci and Ryan [23] identified three factors that improve someone’s well-being: autonomy, relatedness and competence. Intrinsic motivation has an important function for being able to fulfill these three basic psychological needs. The Happiness Route aims to support these needs. Autonomy is central to the intervention, as participants are encouraged to find their own individual passion and act on their own initiative. By activating participants, they often get in contact with others [24], so that the feeling of relatedness will be improved. Finally, participants probably will feel (more) competent when they are conducting an intrinsically motivated activity that fits their individual talents and strengths.

The intervention was invented in the Netherlands in 2006. It has been implemented in ten Dutch cities, many counselors have been trained and over 500 participants followed the Happiness Route. Three pilot studies have shown that the intervention reaches the intended group and is well-received by both counselors and participants [24-26]. A client file analysis showed that the budget was used in a highly functional way and resulted in intrinsically motivated activities, focusing on establishing new contacts and new experiences [24]. Participants retrospectively reported an increase of 40% in well-being and a decrease in consumption of care of 23% [25]. As both the practical experience and pilot studies were promising, it is the right time to start a large and thorough study on the effectiveness of the intervention.

This article thus describes the design of a large evaluation study on the Happiness Route. Based on the pilot studies and practical experiences, we hypothesize that emotional, social and psychological well-being of participants of the Happiness Route will increase in comparison to the traditional and commonly-used problem-based approach and health care will decrease. Furthermore, we will study whether the intervention decreases loneliness, depressive symptoms and consumption of care and whether it increases purpose in life, resilience, social participation and health-related quality. Last, we will examine if characteristics of both participants and counselors have moderating effects. As we handle strict inclusion and exclusion criteria and all counselors will receive the same thorough training, we do not expect moderating effects.

Existing studies on positive psychological interventions mostly had a limited amount of participants, were carried out in experimental settings, and with rather privileged groups with no psychosocial problems, such as students [16]. The present study will belong to the 10% of the largest trials on positive psychological interventions [27]. It is among the first to examine such an intervention in a practice-based research setting with several partners in the field of social work. Our study is also among the first to use an outreaching approach to target individuals with an accumulation of risk factors for low levels of well-being: lower socioeconomic status, social isolation, and health limitations [1-5]. The study will thus provide new insights into the opportunities for mental health promotion in realistic settings.

## Methods and design

### Study design

**Figure 1:**
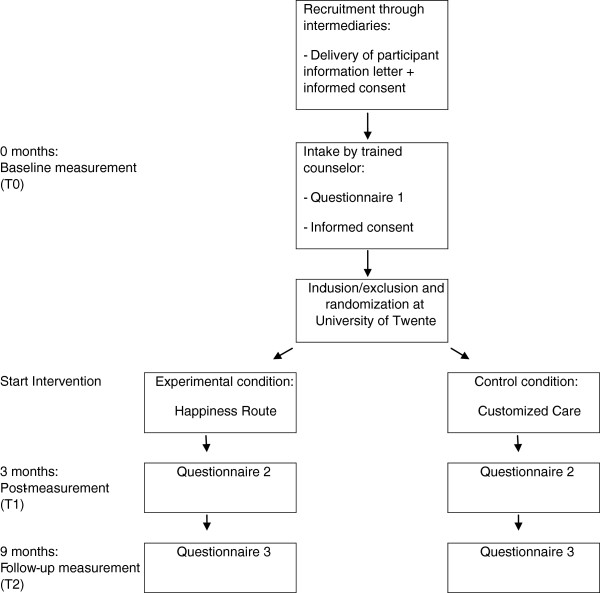
Study flowchart.

To evaluate the effectiveness of the project ‘Happiness Route’, we will carry out a pragmatic multi-site randomized controlled trial with a follow-up at nine months. Participants will be randomly assigned to either the ‘Happiness Route’ intervention or the active control condition ‘Customized Care’, where care will be optimized in two home visits. Measurements will take place at baseline, and three and nine months later. This study has been approved by the Twente Medical Ethics Committee under the file number P12-14 and is registered in the Dutch trial register (3377 NTR). Participation is voluntary and all participants will give written informed consent prior to inclusion. A flowchart of the study can be seen in Figure 1.

### Setting

The study will be carried out in the field of healthcare and social work in nine municipalities in the Netherlands by the University of Twente and Arcon, an organization in the welfare sector. The study is funded by ZonMw (the Netherlands Organisation for Health Research and Development) under the prevention program. A municipal officer will generally be the local project leader, but in some cases, the local project leader works in one of the participating institutions. The local project leader is responsible for the recruitment through intermediaries, the allocation of participants to counselors and for the final approval on spending the budget in the Happiness Route. Municipalities mostly provide financial support for the training of counselors, the appointment of the local project leader, as well as the budget for the Happiness Routes. In other cases, the project is financed by social funds.

The recruitment of participants will be carried out by intermediaries who work as professionals in local care and social work institutions. Examples of intermediary organizations and professions are home care, housing associations, after-care nurses, psychiatric nurses, religious institutions, well-being institutions and organizations for people with mental disabilities. Intermediaries are in direct contact with the target group in their daily work. They can suggest possible candidates to the local project leader, who informed the intermediaries about the study and the target group.

Counselors are experienced professionals, such as social workers, and in some cases experienced volunteers. They are responsible for the intake and the delivery of the experimental and control interventions. To avoid contamination between the experimental and the control conditions, counselors will only deliver interventions in one of these conditions. Both counselors providing the Happiness Route and counselors providing the control condition will receive suitable training.

### Participants

The population in this study consists of adults who are in social isolation, have health problems and a low socio-economic status (SES). Social isolation, poor health and low SES are important correlates of low positive mental health [1-5]. Moreover, they often occur together and mutually influence each other [28-30]. Social isolation is common: 8% of the Dutch adults only have a minimal level of social contacts and 22% feel lonely [31]. Several studies found that loneliness often occurs together with poor income [32-34], health problems [35,36] and low well-being [37]. Socially isolated people with a low SES and health problems are thus an especially vulnerable group with an accumulation of disadvantage.

The following inclusion criteria must all be fulfilled:

– Age: ≥ 18 years

– Social isolation: a score of 3 or higher on the loneliness scale [38];

– Low socioeconomic status (SES): a low educational level (no more than lower secondary education), or a low employment status (no paid employment), or limited financial means, or a combination of these factors. We use the CBS (Statistics Netherlands) budget approach which includes income for basic means as well as participation: € 1000 per month for a single household and € 1370 for a couple or a single parent with one or more children [39];

– Health limitations, i.e., at least one health limitation on the EuroQol [40].

Candidates will be excluded if they fulfill one of the following exclusion criteria:

– High positive mental health: a high score on the Mental Health Continuum-Short Form [41]. To avoid ceiling effects, we use a score of more than one standard deviation above the mean of the Dutch population (i.e., 4.83 or higher) to exclude candidates.

– Serious, untreated clinical depression: a score of 39 or higher on the Center for Epidemiology Depression Scale (CES-D) [42,43].

– Crisis situation: candidates who are in an acute crisis, such as people who have recently lost someone and are still in mourning, who are addicted to alcohol or drugs, or who are homeless. This is judged by the counselor during the intake.

– Insufficient linguistic and cognitive skills to be able to complete the questionnaires, as judged by the counselor during the intake.

### Randomization

When the written informed consent and the baseline questionnaire are received by regular mail, inclusion will be carried out on the basis of the criteria. Each included participant will be appointed randomly to either the intervention or the control condition, based on an a priori computer-generated randomized number list. No stratification will be carried out.

The principal investigator informs the local project leader of the outcomes of the inclusion and randomization procedure, so that the local project leader can appoint a counselor to deliver the intervention in the experimental condition (Happiness Route) or the control condition (Customized Care). It is not possible to blind the conditions to the counselors.

### Experimental condition: the Happiness Route

The intervention builds on the existing intervention Happiness Route but has been formalized and strengthened with the help of existing intervention methods from positive psychology. The goal of the intervention is to promote well-being by means of engagement in intrinsically motivated activities. Methods to promote this are counseling sessions run by trained counselors, based on (positive) psychological interventions. The intervention includes a budget to carry out the activities. The Happiness Route gives participants a financial nudge, i.e., a means to activation, because participants with low socio-economic status tend to have few (monetary) means to engage in activities. The intervention consists of five stages: (1) mutual definition of the situation by the participant and counselor; (2) goal orientation; (3) choice of an activity; (4) planning and carrying out the activity; and (5) early evaluation and feedback in a ‘booster’ session.

Sessions will take place at the home of the participant, with a maximum of 1,5 hours per session. Previous experience has shown that some participants are relatively quick in finding a suitable goal, whereas others need more time and support. Therefore the number of sessions varies between one to four sessions (after the intake), plus the final booster session. The first four stages are flexibly divided over the number of sessions, whereas the booster session always involves early evaluation and feedback between participant and counselor. The aim is to complete the whole process within three months, after which the second measurement will be carried out.

1. Mutual definition of the situation

The first stage involves the definition of the situation. After having looked at health complaints and problems, the problems are put aside. Instead, the participant is motivated to think about life values. The counselor asks the participant questions such as: “What makes you happy?” and “How do you want to live your life?”

2. Goal orientation

The second stage serves to make an inventory of personal goals and possible activities for the Happiness Route. The counselor can make use of methods from existing theoretically based and empirically effective interventions in life review that focus on a productive use of autobiographical memories [44,45]. The participants are asked to recall and name memories from their lives which are particularly important to them. The focus will be on positive memories about activities that made the person happy, engaged, and connected to others. This will be helpful in making a list of possible intrinsically motivated activities that were important in the participants past life. The list can be completed with activities that the participant always wanted to do, like dreams from their childhood and youth, as well as with activities that the participant would like to carry out, independently of memories of past activities.

3. Choice of the activity

The third stage serves to choose an activity. The list that was made earlier can be discussed in terms of opportunities for intrinsic motivation and in being helpful in increasing competence, autonomy, relatedness and well-being. The method of ‘anticipated regret’ is used in order to stimulate a choice that is high on personal relevance and motivation [46,47]: “In a couple of years, which activity would you particularly regret when not carrying it out?” The participant decides which activity will be targeted and how the budget (maximum of €500,-) will be spent. The counselor makes no requirement about the activity, other than that the participant should be intrinsically motivated. A report will be filled out for the local project leader. The project leader decides whether the budget can be transferred to the participant or not. If the project leader is in doubt, the counselor will be asked to spend the next session on a further clarification of how the budget will contribute to the goal engagement of the participant. In a pilot study, it was found that participants spent an average of €422 on activities, focusing on establishing new contacts and going out, as well as learning and making new experiences [24]. Furthermore, many participants chose an activity that challenged their health problem. An example is a former participant with a cognitive impairment, who learned a new language and another participant with a physical impairment, who decided to take swimming lessons. Given the social isolation and health problems, this analysis revealed a highly functional use of the budget [24].

4. Planning and doing the activity

The fourth stage uses the method of behavioral activation from behavioral therapy to help the participant in planning and carrying out the intended activity [48]. The counselor and the participant discuss the steps to be taken and the participants’ behaviors that are needed to actually carry out the activities. The goal of behavioral activation in the present context is to organize positive reinforcement for participants. Socially isolated participants may experience a lack of positive reinforcement in their social environment, due to their lack of contacts. The counselor supports the participants in discovering those reinforcements that the activity is expected to bring.

5. Early evaluation and feedback

The fifth stage is the so-called booster session. During this final session, the participant and counselor evaluate the progress in planning and carrying out the activity. They discuss the benefits of the activities as well as possible pitfalls for continuing the activities.

### Control condition: Customized Care

The goal of the active control condition is to provide the best possible care in the traditional problem-focused manner and to give control subjects attention. This will be done in two home-sessions with a maximum of 1,5 hours per session. After the intake, participants receive two home visits to ensure the optimization of their use of existing care facilities. The approach is the traditional problem-oriented work style. During the sessions, the counselor and the participant work towards a consensual definition of the participant´s (health-related) problems. The counselor and participant then take stock of the use of care and services and the opportunities for optimizing them. The participant is asked how satisfied he or she is with the actual care and services provided and whether there is a mismatch between the problems discussed and the care and services delivered. Does the participant experience problems that are not addressed? Are any care and services redundant, because they do not match the needs of the participant anymore? The counselor and participant then discuss various options for optimizing care and services. As not all participants will know about all services, the counselor informs the participant about all available options to achieve an optimal choice in maintaining, starting or reducing services and helps the participant to realize the optimal choice.

### Measures

#### Data collection

An overview of the measures can be seen in Table 1. Participants have to complete paper and pencil questionnaires during the intake (baseline), after the end of the intervention (three months after the baseline), and nine months after the intake (follow up). All outcome measures will be recorded at all three measurements. Demographic variables will be filled out only at baseline. The baseline questionnaire will be filled out during the intake. Participants fill it out by themselves, but can ask the counselor when help is needed. The second and third questionnaires will be sent by regular mail to participants, accompanied by reply-paid envelopes. If participants need help with filling in the questionnaire, their counselor can help them. Otherwise, the counselor is not involved in the second and third measurement. An overview of the measures can be seen in Table 1.

**Table 1:** Study parameters and measurement instruments

**Outcome measure**	**Instrument**
***Primary outcome measure***	
Emotional, social and psychological well-being	Mental Health Continuum – Short Form (MHC-SF)
***Secondary outcome measures***	
Loneliness	Loneliness Scale
Depression	Center for Epidemiologic Studies Depression Scale (CES-D)
Consumption of care	Items from the Trimbos/iMTA questionnaire for Costs associated with Psychiatric Illness (TIC-P) and use of psychotropic drugs
Purpose in Life	Purpose in Life Scale
Resilience	Brief Resilience Scale
Social participation	Items from different validated survey studies, such as CBS-POLS and LISS
Health-related quality of life	Dutch version of the EuroQol (EQ-5D)
***Moderating measures***	
Demographics of participants	Gender, age, education, marital status, number of children, cultural background, income, employment status
Demographics of counselors	Gender, age, occupational level, financial situation, cultural background
Work characteristics of counselors	Work experience, work conditions
Work engagement	Utrechtse Bevlogenheid Schaal (UBES)
Work satisfaction	Vragenlijst Beleving en Beoordeling van de Arbeid (VBBA), General Work Satisfaction
Adherence of counselors	Online logs

Moreover, Vektis, the Dutch Information Centre for Care, will be asked to provide group statistics about the actual use of care. Vektis registers the use of healthcare by every Dutch citizen. Participants will be asked to provide their health insurance policy number and the name of their health insurance company on the informed consent, together with their permission to use the information as registered by Vektis. Vektis will execute the statistical analyses on possible differences in health care consumption before and after the intervention and to compare the use of care services between the experimental group and the control group.

Counselors also have to fill out a questionnaire before they start with their work for the study. Measures include demographic information, work experience, work conditions, work engagement (UBES; Utrechtse Bevlogenheidschaal) [49] and work satisfaction [50,51]. All counselors have to fill in online logs after each session. Happiness-counselors will give information about the recruitment, note the date of the session, give a short description of the session and describe their impression of the participant. They also have to answer questions about the motivation of the participant, the quality of their relation with the participant, the progress of the participant, the methods used in the session, and the current stage they are at. They also have room for further, free-text comments. The control counselors have to fill out similar, but somewhat shorter logs. After the second session, they have to fill out which changes were initiated, concerning the provision of care.

To assess treatment integrity, counselors will tape a session using a voice recorder. To gain more insight in the evaluation of the Happiness Route from the point of view of participants and counselors, we will conduct interviews with counselors and participants at the end of the study. Counselors and participants will be asked individually whether they want to participate in these interviews.

#### Primary outcome measure

Positive mental health is the primary outcome. It is measured using the Dutch Mental Health Continuum – Short Form (MHC-SF) [41]. The MHC-SF is a 14-item questionnaire that measures three core dimensions of positive mental health [52], which match the three dimensions of the definition of the World Health Organization from 2005 [9]:

• emotional well-being (3 items), defined in terms of the experience of positive feelings and satisfaction with life;

• social well-being (5 items), defined in terms of the self-report of positive functioning in community life (being of social value);

• psychological well-being (6 items), defined in terms of the self-report of positive functioning in individual life (self-realization).

Participants are asked to rate the frequency of feelings they have experienced in the past month. Items are scored on a six-point scale ranging from ‘never’ to ‘every day’. A higher score means more well-being.

Following classical test theory, the three subscales have been found in exploratory and confirmatory factor analyses. The internal consistency, concurrent and discriminant validity of the instrument and its subscales is good. The instrument has satisfactory test-retest reliability, indicating that it is sensitive to change. This is also supported by recent intervention studies [53,54]. A recent analysis based on item response theory shows that the reliability of the individual items is high across demographics, physical and mental health status, as well as across time [55]. A pilot study suggested that there would be no interpretation problems caused by this questionnaire in the target group [24].

#### Secondary outcome measures

1. Loneliness

Loneliness is measured using the eleven-item loneliness scale developed by De Jong Gierveld and Van Tilburg [38]. Loneliness is measured as the subjectively experienced lack of embeddedness in social relations. The experience of loneliness is an inclusion criterion for the study. The intervention targets socially isolated people and aims to ‘nudge’ them out of their isolation. Loneliness is measured to assess if this aim is reached at the level of subjective experiences.

2. Depression

To measure if candidates decrease in depressive symptoms, the Dutch version of the Center for Epidemiological Studies-Depression Scale (CES-D) [42,43] is used. It is also used to test the exclusion criterion that a candidate may not have an untreated serious depression.

3. Consumption of care

Using items from the TIC-P (Trimbos/iMTA questionnaire for Costs associated with Psychiatric Illness) [56], we can assess our hypothesis that the self-reported consumption of care reduces after participating in the Happiness Route. As the outcomes are still self-reported, Vektis, the Dutch information center for care, will carry out analyses on the actual differences in the use of healthcare between the experimental and control group, both before and after the intervention.

4. Purpose in Life

The Purpose in Life Scale [57,58] measures “the feeling that there is a purpose and meaning in life, (…) a clear comprehensibility of life’s purpose, a sense of directedness, and intentionality” [57]. The Happiness Route aims to help participants find a (new) aim and purpose in their lives.

5. Resilience

Resilience is measured using the Brief Resilience Scale [59]. We hypothesize that people who follow the Happiness Route will develop greater resilience. Resilience is often seen as an important individual resource for mental health, especially in difficult situations.

6. Social participation

We measure social participation using items that assess paid or voluntary work, social contacts and activities outside the house. The items are derived from validated national survey studies. The items measure whether the aim of the Happiness Route to nudge people into more social participation is reached.

7. Health-related quality of life

The Dutch version of the EuroQol (EQ-5D) [40] is used to measure if participants will have an improved health-related quality of life after following the Happiness Route. It is also used as an inclusion instrument for assessing whether or not participants have health limitations.

#### Moderating measures

To address the question whether the effect of the intervention is different for participants with varying backgrounds, we will examine whether age, gender, and cultural background moderate the effectiveness of the intervention. To address the question whether the effect of the intervention depends on the characteristics of counselors, we will examine the moderating effects of the characteristics of counselors: work experience, work satisfaction, and adherence. For *work experience* we will compute an index that is based on educational level and number of years’ experience. For *work satisfaction*, we will compute an index that is based on work engagement [49] and job satisfaction [50,51]. Furthermore, counselors will fill out online logs for each participant after every home visit. These logs can be used to measure *adherence*.

### Statistical analyses

#### Descriptive statistics

A CONSORT flow chart of participation during the total study will be drawn. Reasons for drop-out will be summarized. Percentages of missing values and dropout will be displayed. The evaluation interviews with counselors and participants will be subject to a content analysis and the descriptive statistics of the categories resulting from this content analysis will be computed.

#### Univariate analyses

Univariate analyses on the differences between the intervention and control groups at baseline (chi-square and t-tests) will be performed to check whether the randomization has succeeded.

#### Multivariate analyses

We will first do a missing data analysis for all participants on the second and third measurement point. A logistic regression analysis will be conducted with having complete data as the dependent variable, and demographic and psychosocial characteristics at baseline measurement as independent variables to assess non-random missings. We will use multiple imputations (five data sets) to replace missing values and carry out the analyses on the pooled data set. Hence, all participants who were randomized can be included in the statistical analyses. The results from this imputed intention-to-treat sample will be compared to the results of the observed data only.

We will use MANOVA analyses to assess the effectiveness of the intervention in reaching its primary and secondary outcomes. The measures at baseline, post-treatment, and follow-up are repeated measures and the experimental condition versus the control condition is the independent variable. In all analyses, we take into account that the observations are clustered as participants are ‘nested’ in each of the ten local institutions. Therefore, robust standard errors are computed using the first-order Taylor-series linearization method.

Standardized mean differences (Cohen's d) at post-intervention and follow-up are calculated as the difference between the means of the treatment and control condition divided by the standard error of the control condition. We expect a small to moderate effect size for the primary outcome (Cohen’s d= 0.35).

To answer the question on moderating participant factors, we will analyze the moderating effects of demographic characteristics (age, gender, and cultural background) on the effectiveness of the intervention. Following Kraemer et al. [60], we will enter the change score of positive mental health at nine months minus baseline as a dependent variable in a linear regression model. The condition dummy (experimental versus control condition), the potential moderator, and their interaction are independent variables. When the interaction effect is significant, there is a differential effect of the intervention for this particular moderator. Analyses will be carried out for each of the three potential moderator variables separately.

We do similar regression analyses to answer the question on moderating counselor factors. The potential moderators are the indices for work experience, work satisfaction, and adherence to the program as measured with the questionnaire for counselors and the logs of the counselors.

### Power calculation and sample size

Based on a meta-analysis of previous studies in positive psychology, conducted with instruments that are similar to the MHC-SF [27], we expect a small to medium effect size for the intervention Happiness Route (Cohen’s d = 0.35). A pilot study [25] and experiences from previous practice showed that there are no negative effects on the well-being of participants. We therefore specified a one-tailed test with an alpha level of 0.05 under the assumption that the Happiness Route will be more effective than the Customized Care group, as the latter is not intended to improve positive mental health. This has also been shown in previous studies on positive psychological interventions [27]. Given ethical considerations, Knottnerus and Bouter [61] argued that one-sided testing is preferable under these circumstances. Power analysis with the program GPower with a power of 0.80 (1 – beta of 0.20), indicates that 102 participants are needed in each condition. We used a maximal drop-out rate of 20% which is a conservative estimate from previous experience. In everyday practice, the drop-out was about 10% of the more than 500 people who already participated in a Happiness Route. With a maximal drop-out of 20%, we need 128 participants in each condition, 256 in total.

## Discussion

A recently published meta-analysis of RCTs on positive psychology interventions [16] found that, though the interventions can be effective in the enhancement of well-being, the quality of studies was not high. In most studies that examined interventions directed at subjective and psychological well-being, the number of participants was relatively small. Furthermore, the subjects already had a medium to high well-being and no psychosocial problems. A third of the studies included in the meta-analysis were aimed at college students. Only seven of the 38 studies used inclusion criteria to target a group with psychosocial problems. Randomization procedures were often unclear. Also, Bolier et al. stated that statistical analysis could have seriously biased the results, as most studies conducted completers-only analysis instead of intention-to-treat analysis. Many studies took place in experimental settings, so that little is known about their implications and practicability in the field. Most studies described self-help interventions, whereas it was found that individual face-to-face interventions had generally significantly higher effect sizes. Moreover, the control conditions were mostly non-active conditions: only four studies had an active (care-as-usual) control group, whereas 27 studies had either no intervention, a placebo intervention or a waiting list as control condition.

This study tackles the shortcomings named above. First of all, the study belongs to the 10% of the largest studies in the field of positive psychology [16,27]. Secondly, the target group has serious psychosocial problems. Thirdly, randomization procedures are strictly handled and described and an intention-to-treat analysis will be conducted. Furthermore, this is a field study where participants are recruited by referral from practitioners from the health care and social sector. The Happiness Route is not a self-help intervention, as a majority of the positive psychology studies, but is delivered individually and face-to-face to participants. Also, the control condition of this study is an active condition, where the subjects in the control group receive two home visits, to make sure that both groups get attention by a health professional. Furthermore, the control consultant will make sure that the subjects in the control group receive an optimal care-as-usual.

There are several points that make this study especially interesting. This project is directed at an especially vulnerable target group with an accumulation of risk factors: social isolation, poor health and low SES. They are at high risk of suffering from poor well-being. This in turn can have a negative effect on their already poor physical health. This makes this group also a high-cost group in the healthcare sector. The Happiness Route can help to nudge participants towards improved well-being by helping them to find their passion and carrying out an intrinsically motivated activity related to that passion. The outreaching approach to recruit socially isolated participants and the happiness-based working style are innovative ways of approaching and supporting this group. Instead of focusing on their problems and their care, the focus lies on their well-being, connectedness to others, as well as their autonomy and competences. This method is based on the well-proven self-determination theory [6] and effective methods from well-tested interventions are used, such as life review [44,45], anticipated regret [46,47] and behavioral activation [48]. By taking this innovative approach, it is expected that not only will participants’ well-being and psychosocial function improve, but also that their care consumption will decrease.

This is the first study to describe the evaluation of a practice-based program that nudges a target group suffering from health problems and low SES out of their social isolation towards more well-being by a happiness-based approach. Our study will answer questions concerning the effectiveness of this happiness-based approach. We will follow an intervention in practice, from recruitment, intake, and inclusion up to conducting the intervention and measuring effects. We will not only take a closer look at the effects of the Happiness Route, but also study if participants benefit in a similar way. We expect that the inclusion criteria will make sure that the intervention is beneficial to all included participants. We also assume that counselor characteristics will not influence the effectiveness of the intervention, as we train them all in a similar way. To test these assumptions, we will examine the role of characteristics of both participants and counselors. Another strength of this study is the expected high external validity, as the intervention will be examined in a real-life setting and the recruitment for the study is the same as the actual recruitment for the Happiness Route.

However, this RCT also has some limitations. Firstly, the available time to detect a possible change in the outcome measures will be only nine months in total (from intake to follow-up measure) and only six months after the intervention ended. Some changes, especially in the consumption in health care, might only take place later in time, also because institutions are often slow in changing care schemes, such as domestic help. Nine months are however a standard time for follow-up in psychological research. In fact, many studies in the field of positive psychology do not satisfy this standard and use no or shorter follow-up measurements [16].

A further limitation is that the participants in the control condition do not receive any money and will only receive two home visits from a counselor, whereas the participants who follow the Happiness Route will receive three to five home-visits and also will receive a budget. Therefore, it is possible that this extra social and financial attention could be partly an explanation for better effects in the experimental group as compared to the control group. On the other hand, the control group is an active condition where participants will receive home sessions from a trained professional who can help them optimizing their health care, which is better than the often used waiting list condition, in which participants receive no attention at all.

If the intervention has positive effects, we cannot be sure which part of the intervention helped. A positive effect could be due to new social contacts, activation, a new aim and direction in life, a feeling of autonomy or competence or a combination of any of these factors. Thus we can tell if the intervention is effective, but we will not be able to specifically point out which parts of the intervention cause the possible effectiveness. We also will not know if the intervention would also be equally effective with a lesser amount of effort, time and money or which level is enough. However, the current aim is to study the effectiveness of this practice-based intervention. Further studies can clarify which elements and which ‘dose’ of social and financial support will reach the effect.

The study also has to face some challenges. Especially the real life setting is challenging for the internal validity of the study. Different cities have different ideas and requirements about the project, which sometimes conflict with the internal validity of the study. The demands of the study cannot always be met, due to personal circumstances of the counselors. Illness of a counselor can for example cause a delay in the delivery and consequently a delay in filling in the second questionnaire. These circumstances can lead to differences between subjects. Conversely, the results will have more external validity, as the study is performed in a real life setting.

Primarily, the study will evaluate if a happiness-based approach can increase well-being of a vulnerable group. Secondarily, the study will examine if the Happiness Route can decrease loneliness, depressive symptoms and consumption of care and whether it improves purpose in life, resilience, social participation and health-related quality of life. If the hypothesis of the intervention’s superiority over an optimized care as usual approach can be demonstrated, this could convince more communities to use a more happiness-based working style, especially if the study shows that this could result in less health care consumption and thus save money. The results of the study will become available in 2015.

## Abbreviations

MHC-SF: Mental health continuum-short form; SES: Socioeconomic status; RCT: Randomized controlled trial.

## Competing interests

The authors declare that they have no competing interests.

## Authors’ contributions

All authors contributed to the design of the study. LW and GW drafted the manuscript and EB helped to draft this manuscript. GW and EB provided comments. All authors read and approved the final manuscript.

## Authors’ information

GW is Associate Professor in psychology at the University of Twente. He directs the Dutch Life Story Lab (http://www.utwente.nl/lifestorylab) that aims to contribute toward a person-centered approach in mental health care. His work takes place at the intersections of narrative, positive, and lifespan developmental psychology with a particular focus on well-being and positive mental health in later life. EB is full professor *Mental Health Promotion*. His research is mainly directed to design and evaluate technology-supported interventions that improve positive mental health and a person-directed approach in the health care. His research is concerned with positive and narrative psychology, mindfulness, acceptance and commitment therapy and autobiographical reflection. LW is PhD student at the University of Twente at the department Psychology, Health and Technology. In her promotion study she will examine the effects of the Happiness Route on well-being and health care consumption.
